# Supplementary material for: Coenzyme Q10 Supplementation Increases Removal of the ATXN3 Polyglutamine Repeat, Reducing Cerebellar Degeneration and Improving Motor Dysfunction in Murine Spinocerebellar Ataxia Type 3
Source: Nutrients. 2022 Aug 31;14(17):3593. doi: 10.3390/nu14173593 (PMC9459709; doi:10.3390/nu14173593)
Supplement: Supplementary file 1 [file nutrients-14-03593-s001.zip › nutrients-1840677-supplementary.pdf]

nutrients

MDPI

Supplementary Material

Coenzyme Q10 supplementation increases removal of the *ATXN3* polyglutamine repeat, reducing cerebellar degeneration and improving motor dysfunction in murine spinocerebellar ataxia type 3

Yu-Ling Wu [1,2,†], Jui-Chih Chang [3,4,†], Hai-Lun Sun [5,6,†], Wen-Ling Cheng [1], Yu-Pei Yen [7], Yong-Shiou Lin [1], Yi-Chun Chao [8], Ko-Hung Liu [8], Ching-Shan Huang [3], Kai-Li Liu [7,9,*] and Chin-San Liu [1,10,11,12,*]

Supplementary Methods

Treadmill exercise training

12-month-old 84Q SCA3 and CoQ10-treated mice (n = 7 in each group) were randomly assigned to the non-trained (n = 3) and exercise-trained groups (n = 4). The detailed treadmill exercise procedure was used in previous study. The mice were allowed to adapt to their new environment by 1-week familiarization period. In prior to the treadmill exercise training, they ran on a treadmill (model T306, Diagnostic and Research Instruments Co., Taoyuan, Taiwan) for 10 min per day at a speed of 9 m/min. In the experimental period, the animals of exercise-trained group were followed to running for 30 minutes each day at a speed of 12 m/min and 5 times per week for 16 weeks. Mice in the non-trained groups were not subjected to any exercise.

Supplementary Figures

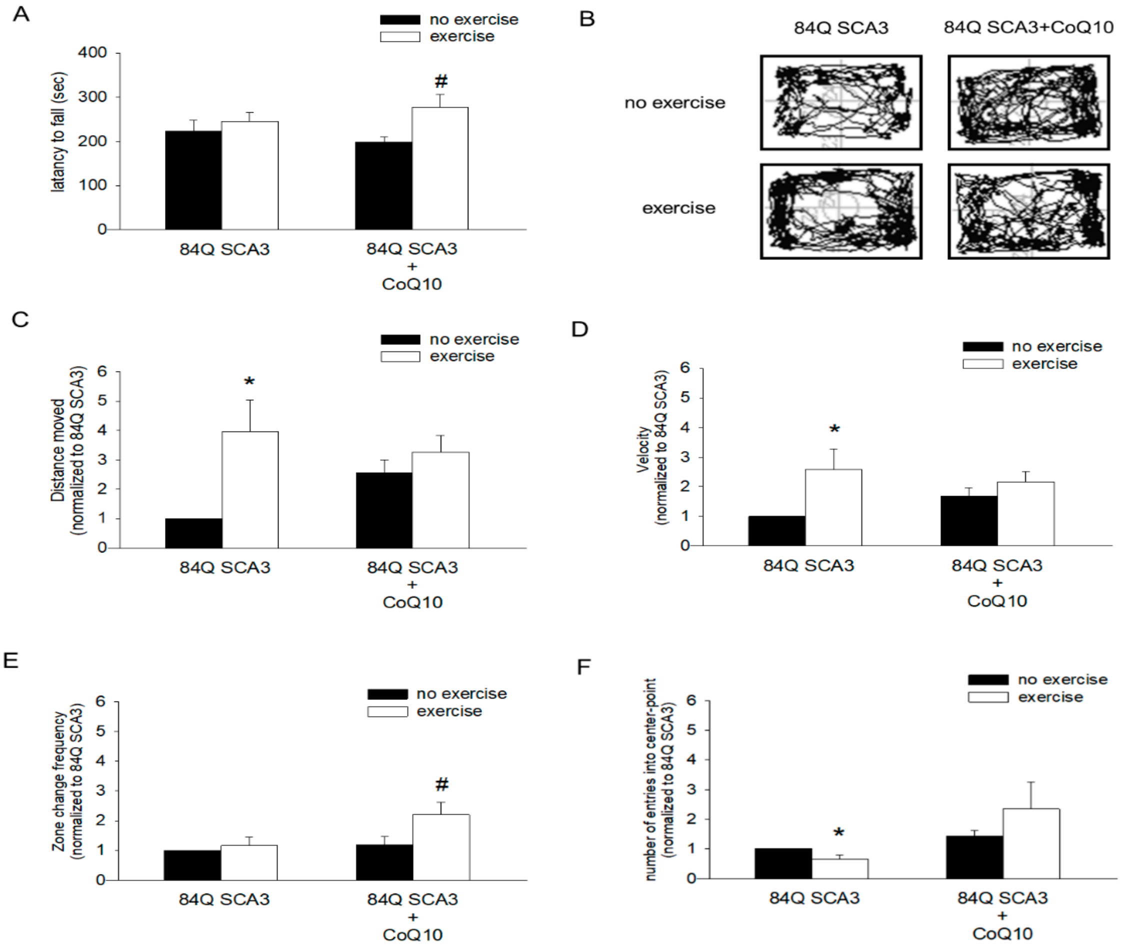

Figure S1. Effects of CoQ10 on motor coordination and locomotive activity after treadmill exercise training in 84Q SCA3 mice. After the age of 12 months, 84Q SCA3 and CoQ10-treated 84Q SCA3 mice were used the treadmill exercise training for 16 weeks. At the age of 16 months of animals, (**A**) we performed an accelerated rotarod test and the latency to fall (seconds that latency time of mice on the rotarod). (**B**) The representative traces of spontaneous movement in all groups of mice by an open field behavior test during a 10-minute monitoring period using overhead tracking system Top Scan. The open field behavior analysis was quantified to attain the (**C**) distance moved, (**D**) average velocity, (**E**) zone change frequency, and (**F**) number of entries into center-point. In (**C–F**) charts, values from the treated mice were normalized to those of the 84Q SCA3 group. Data are presented as the mean ± SD. *$p < 0.05$ indicates exercise group compare to no exercise group in 84Q SCA3 mice. #$p < 0.05$ indicates exercise group compare to no exercise group in 84Q SCA3+CoQ10 mice. 84Q SCA3, chow diet of vehicle; 84Q SCA3+CoQ10, CoQ10 supplementation; no exercise, non-trained treadmill exercise training; exercise, exercise-trained treadmill exercise training.
